# A seven-gene signature predicts overall survival of patients with colorectal cancer

**DOI:** 10.18632/oncotarget.10982

**Published:** 2016-08-01

**Authors:** Huarong Chen, Xiaoqiang Sun, Weiting Ge, Yun Qian, Rui Bai, Shu Zheng

**Affiliations:** ^1^ Cancer Institute (Key Laboratory of Cancer Prevention and Intervention, China National Ministry of Education, Key Laboratory of Molecular Biology in Medical Sciences, Zhejiang Province, China), The Second Affiliated Hospital, Zhejiang University School of Medicine, Hangzhou, Zhejiang, 310009, China; ^2^ Zhong-shan School of Medicine, Sun Yat-Sen University, Guangzhou, 510089, China; ^3^ Department of Gastroenterology, Sir Run Run Shaw Hospital, School of Medicine, Zhejiang University, Institute of Gastroenterology, Zhejiang University, Hangzhou, Zhejiang, 310009, China

**Keywords:** colorectal cancer, gene expression microarray, overall survival

## Abstract

Colorectal cancer (CRC) is a major cause of global cancer mortality. Gene expression profiles can help predict prognosis of patients with CRC. In most of previous studies, disease recurrence was analyzed as the survival endpoint. Thus we aim to build a robust gene signature for prediction of overall survival (OS) in patients with CRC. Fresh frozen CRC tissues from 64 patients were analyzed using Affymetrix HG-U133plus 2.0 gene arrays. By performing univariate survival analysis, 6487 genes were found to be associated with the OS in our cohort. KEGG analysis revealed that these genes were mainly involved in pathways such as endocytosis, axon guidance, spliceosome, Wnt signalling and ubiquitin mediated proteolysis. A seven-gene signature was further selected by a robust likelihood-based survival modelling approach. The prognostic model of seven-gene signature (NHLRC3, ZDHHC21, PRR14L, CCBL1, PTPRB, PNPO, and PPIP5K2) was constructed and weighted by regression coefficient, which divided patients into high- and low-risk groups. The OS for patients in high-risk group was significantly poorer compared with patients in low-risk group. Moreover, all seven genes were found to be differentially expressed in CRC tissues as compared with adjacent normal tissues, indicating their potential role in CRC initiation and progression. This seven-gene signature was further validated as an independent prognostic marker for OS prediction in patients with CRC in other two independent cohorts. In short, we developed a robust seven-gene signature that can predict the OS for CRC patients, providing new insights into identification of CRC patients with high risk of mortality.

## INTRODUCTION

Colorectal cancer (CRC) is one of the most common malignancy with high morbidity and mortality worldwide, accounting for almost 1.4 million new cases and 0.7 million deaths in 2012 [1]. In China, the estimated new cases of CRC were 376,300, with 191,000 deaths occurring in 2015 [2]. Despite of improvements in diagnosis and treatment for CRC, its annual new incidence and mortality is still growing.

CRC is a heterogeneous disease with complex multi-pathways. Most of CRC follows the chromosomal instability (CIN) pathway which is the most well characterized pathway type [3]. The Wnt signaling pathway [4], RAS pathway [5], p53 system [6], and other pathways involved in CIN [7, 8] are frequently present to be dysregulated during the initiation, progression and metastasis of CRC. A number of patients have quite different treatment responses and prognoses although their tumor types are histologically identical. Therefore it is necessary to look deeply into those abnormal molecular pathways and develop individual strategies for CRC diagnosis and therapy. To date, a lot of efforts have been put to identify molecular markers, however, with limited success achieved when focusing on single protein or gene mutation [9]. Gene expression profiling has been verified as a promising tool to classify tumors and predict the prognosis of cancer [10]. Identification of molecular subtypes [11, 12], discovery of progression markers [13, 14], and construction of different prognostic models [15–18] in CRC have been processed and confirmed to potentially improve the diagnosis and therapy of CRC. However, all the developed prognostic signatures are still difficult to apply commonly because of the heterogeneity of CRC.

For most of these studies, disease free survival (DFS) or relapse free survival (RFS) was used as the endpoint to develop molecular markers or to evaluate the validity of prognosis. Considering that overall survival (OS) is traditionally regarded as the ultimate measure of treatment benefits, we would like to investigate whether it is possible to build a robust gene signature to predict the OS in CRC patients. By performing univariate survival analysis, 6487 genes associated with OS were discovered in patients with CRC from a Chinese cohort. Of them, a seven-gene signature was developed using a robust likelihood-based survival modelling approach, and further trained by BRB-array tool to generate a prognostic model. Importantly, the prognostic value of this seven-gene signature was validated in other two independent cohorts, indicating its potential use for identifying CRC patients with high risk of mortality.

## RESULTS

**Figure 1:**
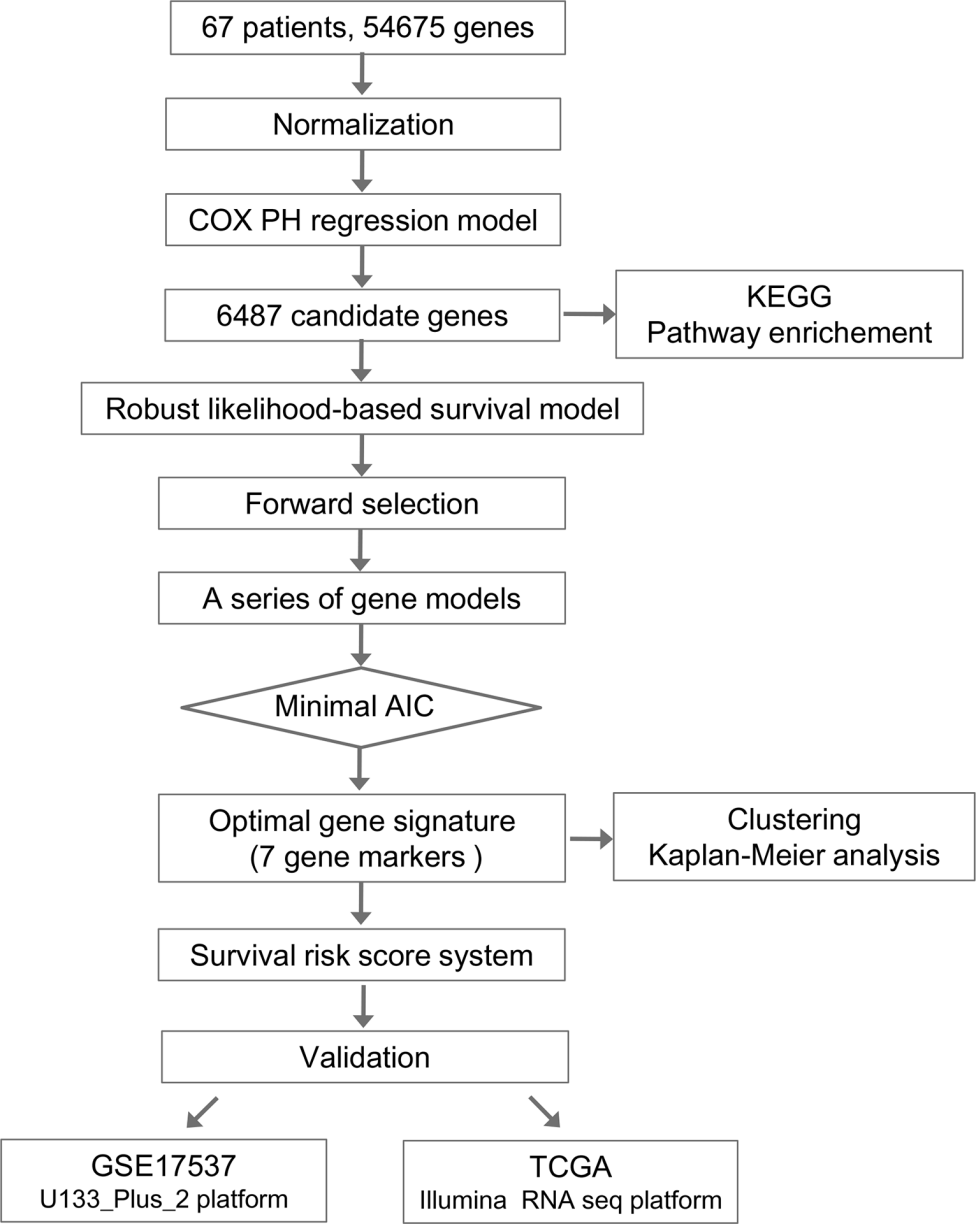
Flow chart of methods for building the seven-gene signature based on 64 Chinese colorectal cancer (CRC) samples Briefly, 6487 genes associated with the overall survival (OS) in CRC patients were first produced by univariate survival analysis. Next a robust likelihood-based survival modelling approach was used to select the optimal gene signature for prognosis prediction. The survival risk score system was built based on seven-gene signature (NHLRC3, ZDHHC21, PRR14L, CCBL1, PTPRB, PNPO, and PPIP5K2), which divided patients into high- and low-risk groups. Finally, this seven-gene signature was validated in two independent cohorts on different platforms.

The overall flowchart of this work was summarized in Figure 1. We employed Cox proportional hazard regression model and forward selection method to identify a seven-gene signature which can predict OS for colorectal cancer patients based on our gene microarray datasets of Chinese patients. To further evaluate the performance of this gene signature, two cohorts on different platforms from other countries were subsequently validated.

### Identification of genes associated with OS

Our data included expression values of 54675 genes and 64 samples. Each sample had observed (survival or censoring) time and censoring status. We first selected an initial set of genes by performing univariate survival analysis using Cox proportional hazard regression model, with the threshold of *p*-value set as 0.05. Total 6487 genes associated with the overall survival were initially identified.

**Figure 2:**
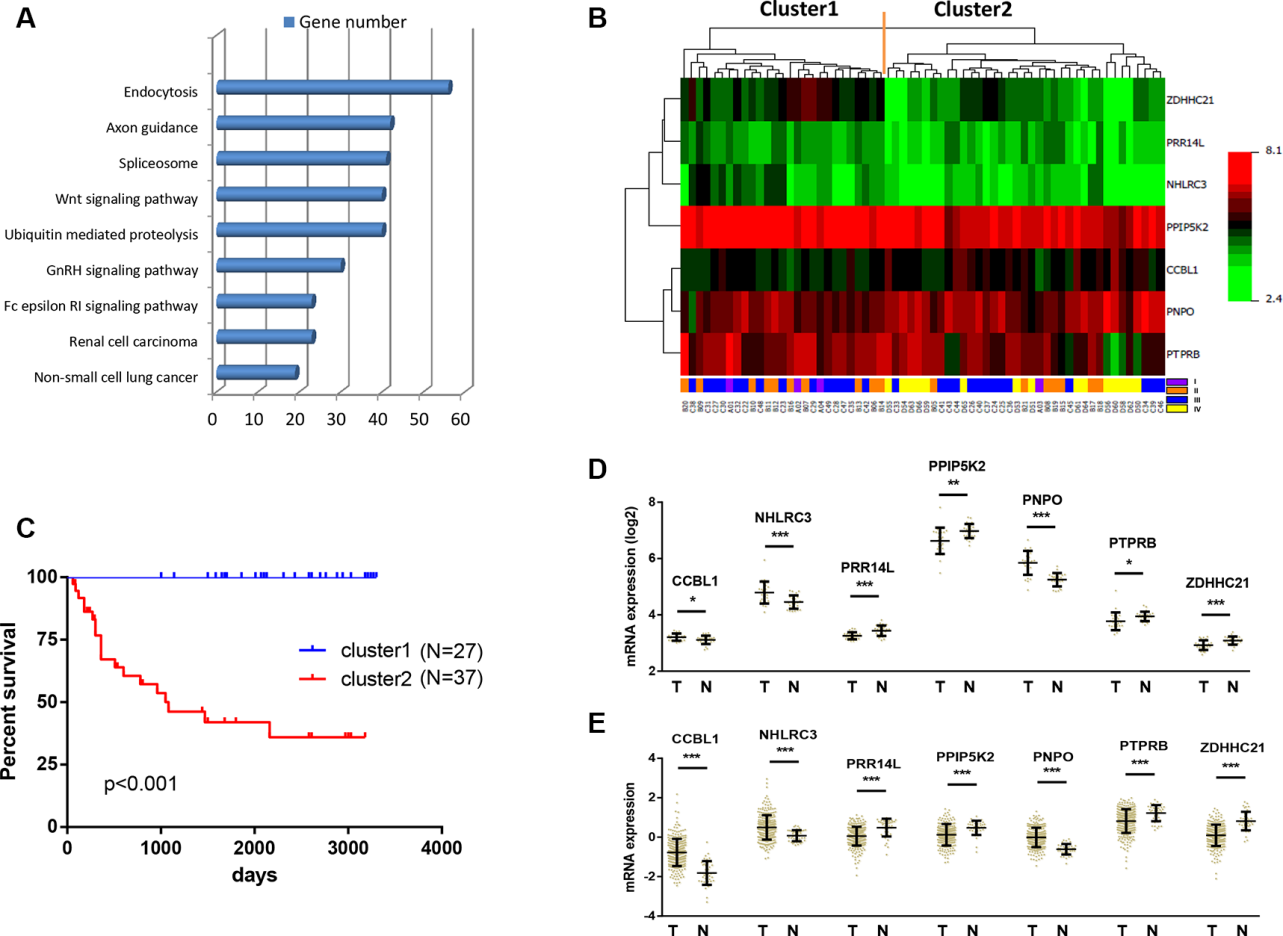
Identification of optimal gene signature for OS prediction (**A**) Enrichment of Kyoto Encyclopedia of Genes and Genomes (KEGG) pathways analysis for 6487 genes associated with the OS. Top 10 pathways were shown. (**B**) The 6487 genes in 64 samples were shown in a heat map (The green and the red colors represent lower and higher expression value, respectively). Unsupervised hierarchical clustering analysis was applied, which divided patients into two clusters. (**C**) Kaplan–Meier curves for patients in different clusters. (**D**) The mRNA expression of CCBL1, NHLRC3, PNPO, PRR14L, PPIP5K2, PTPRB and ZDHHC21 in 26 pairs of CRC tissues and adjacent normal tissues in our cohort. Data is represented by mean ± SD and *p* values were obtained by Wilcoxon matched-pairs test. (*p < 0.05, **p < 0.01, ***p < 0.001). (**E**) The mRNA expression of CCBL1, NHLRC3, PNPO, PRR14L, PPIP5K2, PTPRB and ZDHHC21 in 623 CRC tissues and 51 adjacent normal tissues in TCGA cohort. Data is represented by mean ± SD and *p* values were obtained by Mann–Whitney *U* test. (*p < 0.05, **p < 0.01, ***p < 0.001).

To investigate the key pathways that were associated with patient survival, we next performed KEGG pathway enrichment analysis for the 6487 genes. These genes were found to be enriched in the signalling pathways such as endocytosis, axon guidance, spliceosome, Wnt signalling and ubiquitin mediated proteolysis (Figure 2A).

### Screening of seven-gene signature

We next screened the optimal survival-associated signature genes based on a partial likelihood of the Cox proportional hazard regression model [21]. Considering larger variability of the data, a cross-validation technique was applied by separating the samples into training and validation sets (See Materials and Methods). A forward selection was employed to generate a series of gene models and the optimal model was then selected by using the criterion of minimal AIC (Table 1). Seven genes (NHLRC3, ZDHHC21, PRR14L, CCBL1, PTPRB, PNPO and PPIP5K2) were selected as signature genes that can optimally predict the OS of patients with CRC. With the selected gene signature, unsupervised hierarchical clustering analysis was performed, and the patient population was classified into two sub-classes: Cluster 1 and Cluster 2 (Figure 2B). Compared with Cluster 1 patients, all Cluster 2 patients in this study were still alive during follow-up (Figure 2C). Therefore this seven-gene signature may have important application in predicting the OS for patients with CRC.

**Table 1:** Survival-associated gene signature screening using forward selection

Gene ID	nloglik	AIC	Gene Symbol
236953_s_at	63.51	129.01*	NHLRC3
243835_at	55.24	114.47*	ZDHHC21
236941_at	53.96	113.92*	PRR14L
206037_at	50.62	109.24*	CCBL1
230250_at	49.53	109.06*	PTPRB
222653_at	46.85	105.70*	PNPO
203253_s_at	43.87	101.73*	PPIP5K2
203031_s_at	43.86	103.73	
1557118_a_at	42.42	102.84	
242793_at	42.32	104.65	
205462_s_at	41.73	105.45	
1552319_a_at	40.57	105.15	

### Differential expressions of seven-gene signature in colorectal cancer and adjacent normal tissue

We further compared the expressions of these seven genes between CRC tissues and adjacent normal tissues in two independent data sets. In our cohort, the mRNA expression of CCBL1, NHLRC3 and PNPO were significantly down-regulated in 26 CRC as compared with paired adjacent normal tissues (Figure 2D). On the other hand, PRR14L, PPIP5K2, PTPRB and ZDHHC21 were determined to be over-expressed in CRC (Figure 2D). Consistent result was obtained using TCGA cohort that contained 623 CRC and 51 adjacent normal tissues (Figure 2E). Therefore, these seven genes may play important roles as oncogenes or tumor suppressor genes during the development of CRC.

### Construction of survival risk score system based on seven-gene signature

**Figure 3:**
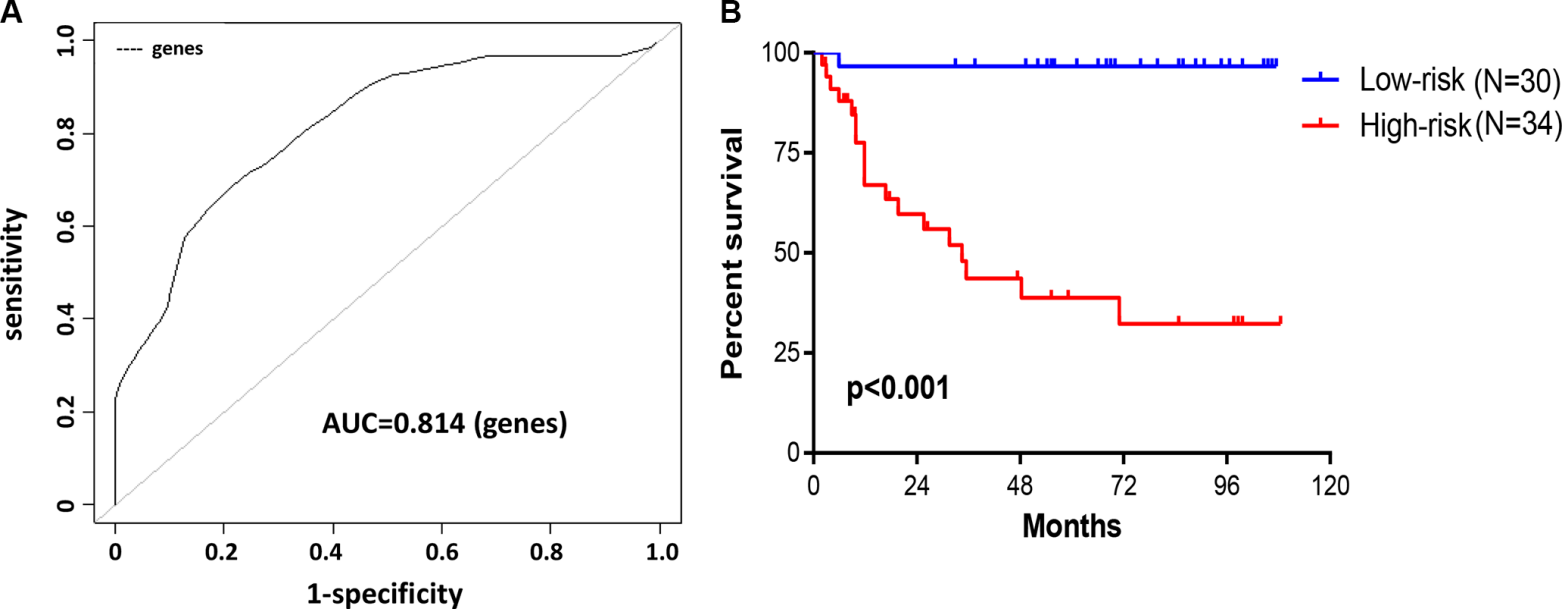
Construction of survival risk score system based on seven-gene signature (**A**, **B**) A prognostic model was developed in BRB-ArrayTools software using penalized Cox regression of the seven-gene signature. Leave-one-out cross-validation (LOOCV) was employed to evaluate the accuracy of predicting OS in 64 CRC patients. (A) The cross-validated time-dependent ROC curve was generated for survival predictions with an AUC of 0.814. (B) Patients were divided into high- and low-risk groups by cross-validated Kaplan–Meier curve.

We subjected these seven genes to BRB-arrayTools to construct a survival risk score system by using the 64 training samples. The regression coefficient for each gene was generated. The survival risk score was calculated as follows: risk score = (–1.004× expression level of PPIP5K2) + (0.823× expression level of CCBL1) + (–0.715× expression level of PNPO) + (–0.008× expression level of PTPRB) + (–1.077× expression level of PRR14L) + (–0.133× expression level of NHLRC3) + (–0.781× expression level of ZDHHC21). Higher score indicated greater mortality risk for patient with CRC. By performing cross-validated time-dependent ROC curves, the area under the respective ROC Curves (AUC) was 0.814 (Figure 3A), confirming the prediction accuracy of this model. 64 patients were divided into high- and low-risk groups. The OS for patients in high-risk group was significantly poorer compared with patients in low-risk group (hazard ratio (HR) = 25.79, *P* < 0.001) (Figure 3B).

### External validation of seven-gene signature

**Figure 4:**
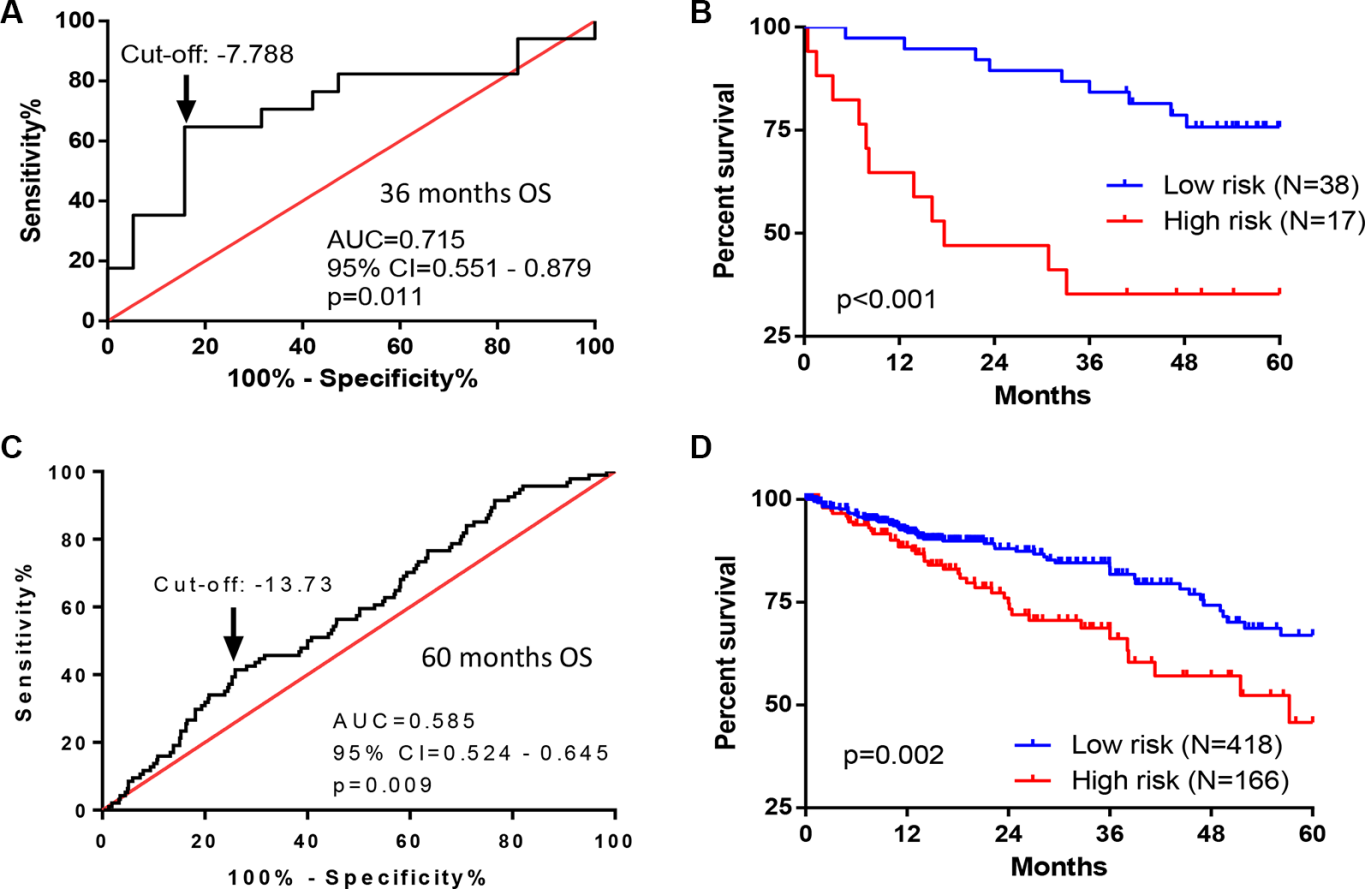
Performance of seven-gene signature in predicting OS in CRC patients from two independent cohorts (**A**, **B**) ROC and Kaplan–Meier curves for the seven-gene signature in dataset from Vanderbilt Medical Center (GSE17537, *N* = 55). (A) The ROC curve was generated for 3-year overall survival predictions with an AUC of 0.715 (*p* = 0.011). Optimal cut-off value (–7.788) was obtained to divide the patients into low and high risk groups. (B) Patients in high risk group had poorer OS as compared with patients in low risk group (HR = 4.475, *p* < 0.001). (**C**, **D**) ROC and Kaplan–Meier curves for the seven-gene signature in the TCGA dataset (*N* = 584). (C) The ROC curve was generated for 5-year overall survival predictions with an AUC of 0.585 (*p* = 0.009). Optimal cut-off value (–13.73) was obtained. (D) Patients in high risk group had poorer OS as compared with patients in low risk group (HR = 1.915, *p* = 0.002).

To evaluate the robustness and effectiveness of the seven-gene signature, we used two independent sets of CRC patients with OS information and gene expressions on different platforms. The first one was from the Vanderbilt Medical Center (GSE17537, *N* = 55) using the same gene expression array platform as we did. The second one, TCGA dataset (*N* = 584), used Illumina RNA Sequencing method. The survival risk score of each patient was calculated based on expressions of seven-gene signature. ROC curve analyses demonstrated that this seven-gene signature was capable of predicting OS in patients with CRC (for cohort I, AUC = 0.715, *p* = 0.011; for cohort II, AUC = 0.585, *p* = 0.009) (Figure 4A and 4C). We further divided the patients into two risk groups, based on optimal cut-off risk scores (Figure 4A and 4C). For the first validation data set, 38 (69.1%) and 17 (30.9%) patients were distinguished as the low and high risk groups, respectively. For the second validation data set, 418 (71.6%) and 166 (28.4%) patients were classified as low and high risk groups, respectively. Kaplan-Meier plots indicated significant differences between 5-year OS of two groups in both two validation data sets: GSE17537 (*p* < 0.001, Figure 4B), and TCGA (*p* = 0.002, Figure 4D). By univariate (Table 2) and multivariate analyses (Table 3), this seven-gene signature significantly predicted the 5-year OS of patients. In multivariate analyses, this seven-gene signature showed prognostic significance for 5-year OS risk in both two validation data sets: GSE17537 (*p* = 0.002) and TCGA (*p* = 0.005). Therefore, the seven-gene signature was an independent prognostic factor in predicting the OS of patients with CRC.

**Table 2:** Univariate Cox regression analysis of potential prognostic factors for patients with CRC

Cohort I				Cohort II			
Characteristics	No. of Patients	5 yr SR (%)	*p* value	Characteristics	No. of Patients	5 yr SR (%)	*p* value
Age			0.311	Age			0.042
< = 60	25	72.0%		< = 60	190	90.0%	
> 60	30	56.7%		> 60	394	81.0%	
Gender			0.395	Gender			0.534
Female	29	58.6%		Female	267	84.6%	
Male	26	69.2%		Male	317	83.3%	
Pathologic_stage			0.100	Pathologic_stage			< 0.001
I, II	19	78.9%		I	103	96.1%	
				II	210	89.5%	
III, IV	36	55.6%		III	172	82.0%	
				IV	85	62.4%	
Risk			0.001	Risk			0.002
Low	38	76.3%		Low	418	86.8%	
High	17	35.3%		High	166	76.5%	

**Table 3:** Multivariate Cox regression analysis of potential prognostic factors for patients with CRC

Cohort I					Cohort II				
Characteristics	Exp(B)	95.0% CI for Exp(B)		*p* value	Characteristics	Exp(B)	95.0% CI for Exp (B)		*p* value
		Lower	Upper				Lower	Upper	
Age					Age				
< = 60	1.000 (ref)				< = 60	1.000 (ref)			
> 60	1.836	0.718	4.694	0.204	> 60	2.384	1.367	4.158	0.002
Gender					Gender				
Female	1.000 (ref)				Female	1.000 (ref)			
Male	0.908	0.361	2.286	0.838	Male	0.991	0.648	1.517	0968
Pathologic_stage					Pathologic_stage				
I, II	1.000 (ref)				I	1.000 (ref)			
					II	1.950	0.670	5.677	0.221
III, IV	2.382	0.748	7.591	0.142	III	4.418	1.554	12.562	0.005
					IV	11.795	4.166	33.396	< 0.001
Risk					Risk				
Low	1.000 (ref)				Low	1.000 (ref)			
High	4.062	1.646	10.027	0.002	High	1.833	1.196	2.810	0.005

**Figure 5:**
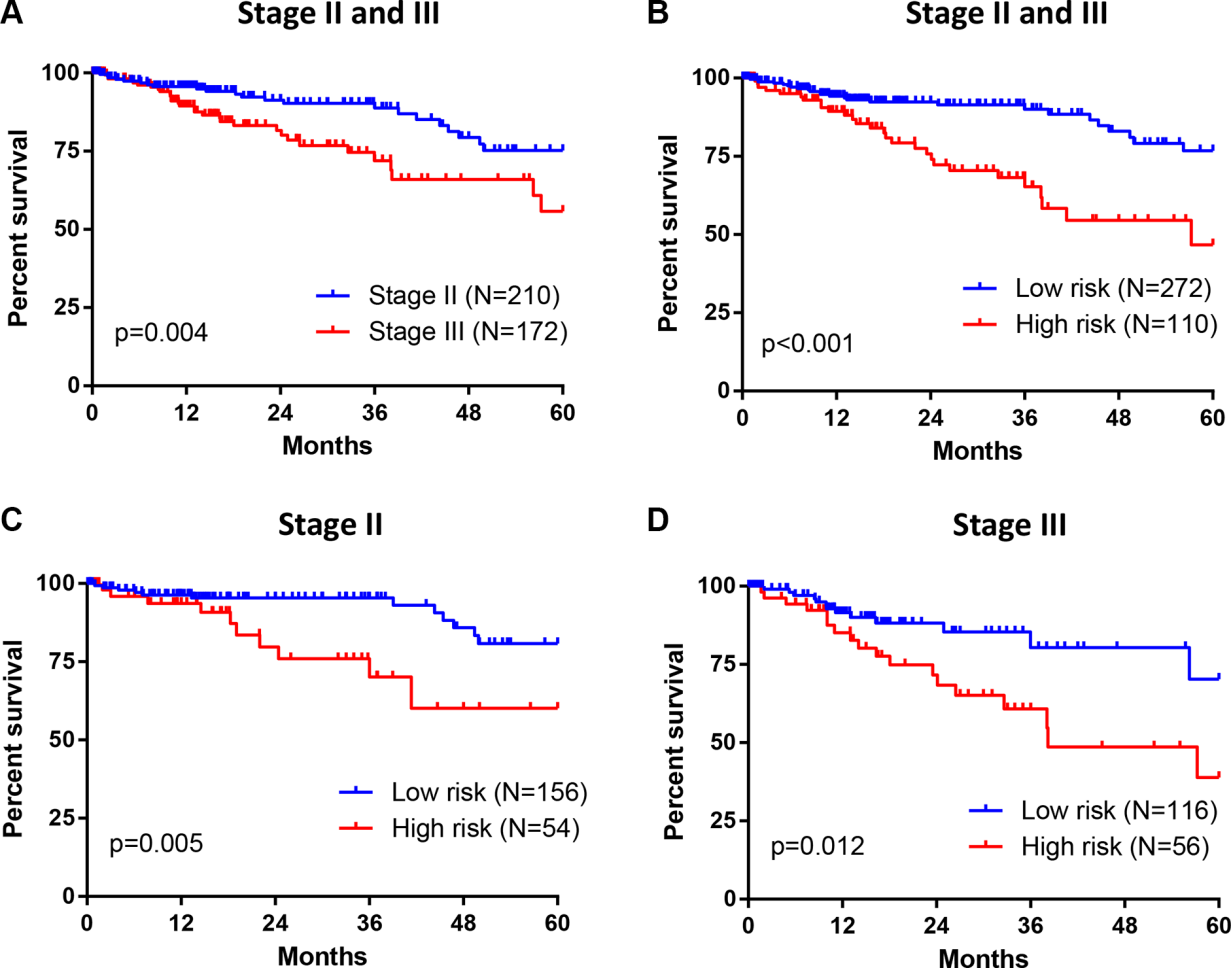
Association between seven-gene signature and OS in patients with stage II and III CRC (**A**) For 382 CRC patients from TCGA, Stage III tumors were associated with greater 5-year mortality than stage II tumors. (**B**) 382 CRC patients were divided into high and low risk group based on their survival risk scores. The association between seven-gene signature (risk scores) and 5-year OS in patients with stage II and III CRC was assessed simultaneously (panel B) or individually (stage II in panel (**C**); stage III in panel (**D**) by Kaplan–Meier curves.

To evaluate this seven-gene signature with respect to the prognosis among patients in TNM stage II and III, we further investigated the association between risk scores and OS in 382 CRC patients from TCGA. Stage III tumors were associated with a higher rate of 5-year mortality than stage II tumors (HR = 2.160, *p* = 0.004) (Figure 5A). Meanwhile, patients in high risk group demonstrated poorer survival as compared with patients in low risk group (HR = 2.918, *p* < 0.001) (Figure 5B). Moreover, this increased 5-year mortality in high risk group was marked in both stage II (HR = 3.124, *p* = 0.005) (Figure 5C) and stage III (HR = 2.415, *p* = 0.012) (Figure 5D). Therefore, this seven-gene signature might be able to help predict prognosis for patients with stage II and stage III CRC.

## DISCUSSION

In this study, we presented the development and validation of a robust seven-gene signature which was able to predict the OS for CRC patients. The signature identified patients with high risk of mortality who may need potential interventions and individual therapies.

A Chinese cohort containing 64 CRC patients was used and total 6487 genes were identified to be associated with the overall survival. Of them, several genes have been reported to present prognostic value for CRC patients, such as CDX2 [19], EPHA2 [20], SLC2A1 [21], CDKN2A [22] and ITGA3 [23]. By performing KEGG analysis, these genes were enriched in the signalling pathways such as endocytosis, axon guidance, spliceosome, Wnt signalling and ubiquitin mediated proteolysis (Figure 2A). The activation of the Wnt signalling pathway is a critical event which frequently occur during CRC development, making it as therapeutic targets for cancer therapy [24]. The ubiquitin-proteasome system is important for cell growth and apoptosis regulation, making it also a potential molecular target for cancer treatment and prevention [25]. We further narrowed down the genes size and selected an optimal seven-gene signature (NHLRC3, ZDHHC21, PRR14L, CCBL1, PTPRB, PNPO and PPIP5K2) for prognosis prediction. By searching NCBI (http://www.ncbi.nlm.nih.gov/gene/), none of these seven genes have been reported previously in cancer study. Of note, NHLRC3 was predicted to play role in protein modification process through ubiquitination; CCBL1, PNPO, PPIP5K2 and ZDHHC21 were possibly involved in cellular metabolism regulation or acting as cell signaling molecules; PTPRB is a member of the protein tyrosine phosphatase (PTP) family which is known to regulate a variety of cellular processes including oncogenic transformation [26]; the function of PRR14L remains unknown. Interestingly, we found that these genes were differentially expressed in CRC tissues compared with adjacent normal tissues in two different cohorts (Figure 2D and 2E). This suggests that these genes might be novel oncogenes or tumor suppressor genes, and that their functions need further investigation.

The prognostic signature was validated in two independent patient sets on different platforms from different countries, with further validation study from China currently underway. Indeed, By ROC curve analyses, this seven-gene signature was capable of predicting OS in patients with CRC from these two datasets with an AUC of 0.715 and 0.585, respectively (Figure 4A and 4C). By multivariate analyses, this seven-gene signature was validated as an independent prognostic factor for predicting 5-year OS of CRC patients (Table 3). Comparing with other studies that constructed different prognostic models [15–18], our study obtained a signature with few genes (only seven) to successfully predict the OS in CRC patients. This is important to develop a RT-PCR assay for clinical practice. In our future study, the high-risk patients identified by seven-gene signature will be further studied for their responses to different therapies in an independent Chinese cohort.

The American Joint Committee on Cancer (AJCC) TNM staging system is a golden standard to determine the treatment and prognosis for patients with CRC. However, the limitation of TNM staging system to accurately predict prognosis has been also demonstrated in clinical practice. For example, the 5-year survival rate of stage IIIA patients (85.4%) was higher than stage II patients (79.2%) [27]. Increasing attention has been focused on patients with stage II and stage III CRC, as the use of adjuvant chemotherapy to a proportion of these patients may cause potential under treatment or overtreatment [28–30]. To date, there are several tests (such as Oncotype Dx [31], a 12-gene RT-PCR assay, and Coloprint, an 18-gene microarray [32]) being developed in clinical laboratories, which are intending to predict the prognosis of patients with stage II CRC and help guide the use of chemotherapy. Still, all of them are under evaluation in multiple validation cohorts. Our seven-gene signature is able to predict the OS for patients in stage II and III in TCGA cohort (Figure 5). However, the sample size is limited. Hence large samples are required to be collected in future to further validate the prognostic value of this seven-gene signature on CRC patients in stage II and III. Moreover, we will study whether the seven-gene signature could benefit selection of adjuvant chemotherapy for individual patient in stage II and III.

In conclusion, our seven-gene signature provides new promising biomarkers for CRC prognosis and potential therapeutic targets for CRC treatment.

## MATERIALS AND METHODS

### Human CRC Samples

Frozen tumor specimens (*n* = 64) and paired adjacent non-tumor tissue (*n* = 26) of 64 patients with colorectal cancer were retrieved from the Second Affiliated Hospital of Zhejiang University, College of Medicine, China. All patients who received surgical operation and were diagnosed as colon or rectal adenocarcinoma were included in this study. All pathologic information and follow-up data were obtained by reviewing the hospital records [33]. Notably, 19 of the 64 patients died of cancer during follow-up. The clinical data of patients was presented in Table 4. This study was approved by the ethics committee of Zhejiang University.

**Table 4:** Patient characteristics

Variables	Case number (*N* = 67) *N* (%) or mean (range)
Gender	
Male	41 (61.2%)
Female	26 (38.8%)
Age (years)	
Male	61 (36–92)
Female	54.5 (19–83)
TNM stage (T)	
T1	1 (1.5%)
T2	6 (9.0%)
T3	35 (52.2%)
T4	25 (37.3%)
TNM stage (N)	
N0	25 (37.3%)
N1	25 (37.3%)
N2	17 (25.4%)
TNM stage (M)	
M0	49 (73.1%)
M1	18 (26.9%)
TNM stage	
I	4 (6.0%)
II	17 (25.4%)
III	28 (41.8%)
IV	18 (26.9%)

### Gene expression microarray and data analysis

Fresh CRC samples were immediately snap frozen in liquid nitrogen and stored at –80°C until further use. Total RNA was extracted using Trizol reagent (Invitrogen, Carlsbad, California, USA) and sent out to company (Capitalbio Corporation, Beijing, China) for RNA quality control (QC) performance. Only high quality RNA (OD260/280 = 1.8~2.2; 28S:18S ≥ 1.5; RIN (RNA Integrity Number) ≥ 8) were used for Affymetrix HG- U133plus 2.0 gene array. Statistical analyses of microarray data were performed in the R language environment (http://www.r-project.org) [34]. The data was normalized and transformed to expression values by using justRMA( ) function in “affy” library. Signal values for all genes were transformed to the log base 2. Quantile normalization was applied to obtain equal distributions of the probe signal intensities.

### Gene signature identification

A robust likelihood-based survival modeling approach [35] was used to select the gene signature. We implemented our analysis by using the “rbsurv” package in R. The detailed algorithm is summarized as follows:

(i). We randomly divided the samples into the training set with N*(1 − p) samples and the validation set with N*p samples, with *p* = 1/3. We fitted a gene to the training set of samples and obtained the parameter estimate for this gene. Then we evaluated log likelihood with the parameter estimate and the validation set of samples. This evaluation was repeated for each gene.

(ii). We repeated the above procedure 10 times, thus obtaining 10 log likelihoods for each gene. The best gene, g(1), with the largest mean log likelihood was selected.

(iii). We searched the next best gene by evaluating every two-gene model and selected an optimal one with the largest mean log likelihood.

(iv). We continued this forward gene selection procedure, resulting in a series of models. Akaike information criterions (AICs) for all the candidate models were computed and an optimal model with the smallest AIC was selected finally.

### Unsupervised hierarchical clustering and Kaplan-Meier analysis

Unsupervised hierarchical clustering analysis [36] was performed in R using “hclust” function with Euclidean distance. Kaplan-Meier curves for two distinct groups of patients were plotted using “survfit” function in survival package. *P* value from log rank test was computed using “survdiff” function.

### Development and validation of 7-gene survival risk score system

We subjected the seven genes to BRB-array Tools (http://linus.nci.nih.gov/BRB-ArrayTools.html), using survival risk group prediction tool [37], to calculate the regression coefficient for each gene using the 64 training samples from Zhejiang University. The Survival Risk Score is the sum of the product of the expression level of a gene and its corresponding regression coefficient. The patients were divided into two groups at high and low risk using the 50th percentile. Leave-one-out cross-validation method was performed to determine the robustness.

Gene expression data from the Vanderbilt Medical Center (GSE17537, HG-U133_Plus_2 platform; *N* = 55) with overall survival information was normalized by justRMA( ) and used as the first validation data set [14]. The CRC dataset (*N* = 623 for primary CRC tumors and *N* = 51 for adjacent non-tumor tissues) from The Cancer Genome Atlas (TCGA, http://cancergenome.nih.gov/; Illumina GA or Hiseq platform) was analyzed (publicly available TCGA pre-processed data was downloaded from Broad GDAC Firehose (https://gdac.broadinstitute.org/)), and those with overall survival information (*N* = 584) were used as the second validation set. For each independent cohort, the risk score of patients was calculated using the coefficient derived from the training data set. Receiver operating characteristic (ROC) curve analyses were generated for the 3- and 5-year survival predictions to evaluate the specificity and sensitivity of the gene signature and to estimate discriminatory power of the prognostic gene expression signatures. The area under the curve (AUC) was calculated and a bootstrap method was used to calculate the 95% confident internal (CI) for AUC. Optimal cut- off value based on ROC curve was obtained to divide the patients into low and high risk groups [38]. Kaplan-Meier curves for the two groups of patients were plotted using “survfit” function in survival package.

### Enrichment analysis of kyoto encyclopedia of genes and genomes pathways

Genes that were associated with the overall survival of patients evaluated by Cox proportional hazard regression model (*P* < 0.05) were included in the Kyoto Encyclopedia of Genes and Genomes (KEGG) pathway enrichment analysis using the Gene Set Analysis Toolkit V2 (http://bioinfo.vanderbilt.edu/webgestalt/). The hypergeometric test statistical method and the BH multiple test adjustment method were used. All genes from human beings were used as reference. Top 10 pathways with at least 10 genes involved were considered significantly enriched.

### Statistical analysis

Mann–Whitney *U* test and Wilcoxon matched-pairs test were performed to compare the gene expression between CRC and adjacent normal tissues. All statistical tests were performed using Graphpad Prism 5.0 (GraphPad Software Inc, San Diego, CA, USA), and a 2-tailed *P* value of less than 0.05 was considered statistically significant
